# Acquired segmental sigmoid hypoganglionosis: A case report

**DOI:** 10.1097/MD.0000000000018803

**Published:** 2020-01-24

**Authors:** Zhi-Ping Pan, Lu-Qiao Huang, Jun-Hui Cui

**Affiliations:** a The Second Clinical Medical College, Zhejiang Chinese Medical University; b Department of Anorectal Surgery, Tongde Hospital of Zhejiang Province, Hangzhou, China.

**Keywords:** acquired hypoganglionosis, pathological inflammatory, surgery, transition zone

## Abstract

**Rationale:**

Intestinal hypoganglionosis most commonly presents in infancy or childhood, with only a few cases reported in adults. Those are mainly diagnosed after elective surgery for long-standing constipation and megacolon.

**Patient concerns:**

We report a case of a 48-year-old female from China who presented with symptoms of discontinuation of bowel movements for 2 months. A hard, round mass could be felt in her right lower abdomen.

**Diagnosis:**

The following examination methods diagnosed acquired segmental sigmoid hypoganglionosis. An abdominal computed tomography revealed a dilatation of the colon and suspicious wall thickening of the sigmoid colon. Anorectal manometry revealed relaxation of the anal sphincter. Histological examination revealed lower numbers and the degeneration of ganglion cells.

**Interventions:**

Sigmoidectomy and transverse colostomy.

**Outcomes:**

The patient recovered well from surgery. Three months after the surgery, barium enema revealed a recovery in colorectal dilatation.

**Lessons:**

This case could help raise awareness of acquired segmental hypoganglionosis. Resection of TZ and enterostomy presents an effective remission strategy for patients at risk of anastomotic leakage due to poor intestinal conditions.

## 1 Introduction

Hypoganglionosis (HG) is a rare condition that is characterized by a reduced density of enteric ganglion cells. Clinically, the symptoms of HG are similar to those of Hirschprung disease (HD), but the presence of ganglion cells can distinguish the 2 diseases.^[1]^ HG can be divided into 2 different subgroups (type I and type II).^[2]^ based on the presence or absence of a locally narrowed transition zone (TZ). HG usually occurs at infancy or in childhood. In congenital HG, the number and size of ganglion cells decrease at birth. Acquired HG (adult-onset form) is extremely uncommon and causes intractable constipation or pseudo-obstruction. Histologically, it is characterized by ganglion cell degeneration and gliosis.^[3]^

## 2 Case presentation

A 48-year-old female Chinese patient was admitted to our department for discontinuation of bowel movements for 2 months. The patient reported the alternation of constipation and diarrhea after an abdominal mass was found 3 years earlier. No laxatives or illicit substances had been used. She had chest tightness and shortness of breath. However, there were no signs of intestinal obstruction, including vomiting or abdominal pain. Family history was unremarkable. On examination, the abdomen was grossly distended, and a hard, round mass was found in the right lower abdomen. No peritoneal irritation was found, and rectal examination revealed an empty rectum.

**Figure 1:**
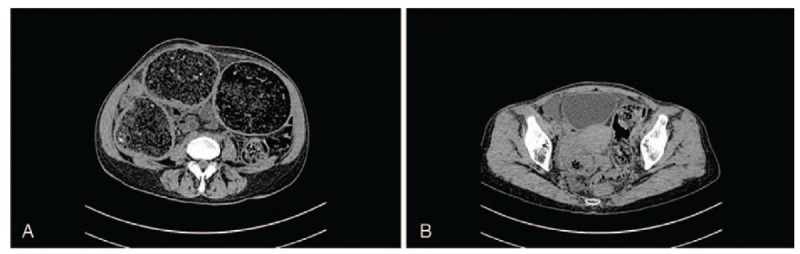
Computed tomography: dilatation of the colon above the sigmoid colon.

The carbohydrate antigen 125 level in the blood was 91 U/mL, and the hemoglobin was 103 g/L. The serologic test for syphilis was positive. Abdominal computed tomography (CT) revealed fecal accumulation and dilatation of the colon above the sigmoid colon and suspicious wall thickening of the sigmoid colon (Fig. 1). Anorectal manometry revealed relaxation of the anal sphincter.

**Figure 2:**
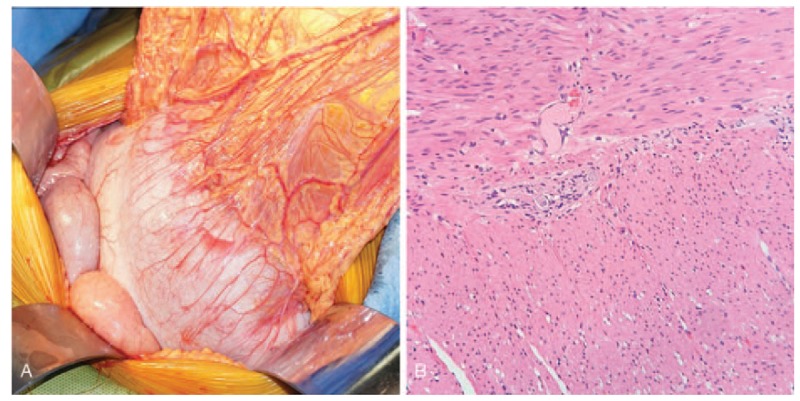
(A) Intraoperative findings: dilated transverse colon. (B) Histology findings: myenteric plexus lacking ganglion cells (hematoxylin and eosin staining, magnification 20×).

Conservative treatment was ineffective, and an urgent surgical intervention was planned. The laparotomy revealed a significantly dilated transverse and descending colon (Fig. 2A) and the sigmoid colon was redundant and narrow. The transverse colon was cut open, and a drainage tube was placed to flush intestinal feces. We excised the sigmoid colon partially and performed a transverse colostomy.

The postoperative period was uneventful. Histological examination of the sigmoid colon showed chronic inflammation of the intestinal mucosa and a decreased number and the vacuolar degeneration of ganglion cells in the myenteric plexuses, on average more than 4 cells/cm (Fig. 2B). Inflammatory cells had infiltrated the serosa, and the blood vessels were dilated and bruised. Immunohistochemistry showed the tissue was positive for soluble protein-100 (S-100), synaptophysin, and neuron-specific enolase. The patient did not have a history of chronic constipation or pseudo-obstruction, and was diagnosed as having acquired segmental HG. Since there was focal stenosis of the sigmoid colon, the condition was typed as type I HG.

**Figure 3:**
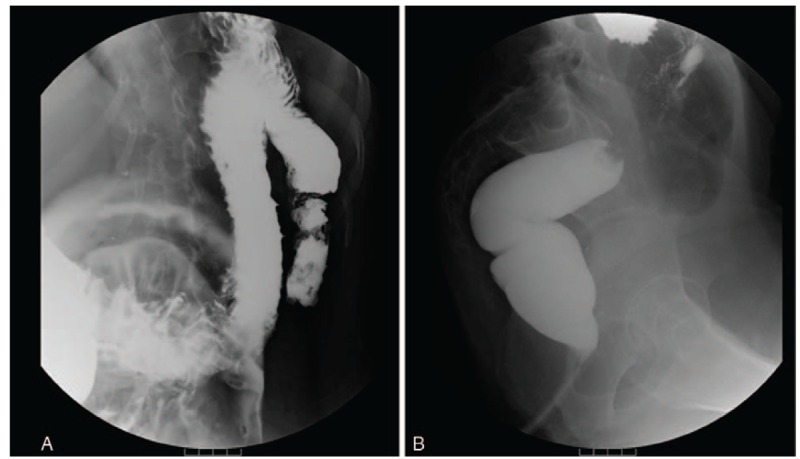
Barium enema: recovery of the colorectal shape.

Three months after the surgery, a barium enema revealed a recovery in colorectal dilatation (Fig. 3). Since her discharge following surgery, the feces at the colostomy were excreted unobstructed and the abdominal volume was significantly reduced. Chest tightness and shortness of breath had disappeared.

## 3 Discussion

HG is a rare condition that accounts for only 5% of neuronal intestinal malformations.^[4]^ The diagnosis of HG is only possible by histochemical examination of a full-thickness biopsy. The histopathological characteristics include a significant reduction in the number of ganglion cells, thickened muscularis mucosae and muscularis propria layers, and a decrease in the activity of acetylcholinesterase in the lamina propria. More recently, S-100 and peripherin were validated as a valuable tool for the diagnosis of HG.^[5]^ On double-contrast barium enema and CT images in adult HG, the proximal colonic segment is markedly dilated with a TZ, and the distal colonic segment becomes narrowed. Compared with HD, the TZ ratio (dilated colon to narrowed colon diameter ratio) was found to be lower in HG.^[6]^

In congenital HG, ganglion cells are small in size and limited in number at birth. The size tends to increase over time, but the number of cells does not change. Acquired HG is delayed, and its histological characteristics are degeneration of ganglion cells and glial degeneration.^[3]^ Compared with the high incidence of congenital HG, acquired HG is extremely rare. Taguchi et al,^[7]^ confirmed 9 cases of acquired HG in a 10-year analysis from 2001 to 2010 in Japan. With or without a narrowed segment with a diminished number of ganglion cells, HG patients can be classified into 2 subgroups. Type I (focal type) has focally narrowed TZ with few ganglion cells (mean 4.9 ± 5.3 cells/cm) that results in functional obstruction. Type II (diffuse type) shows a diffuse reduction in ganglion cell numbers (mean 13.4 ± 3.7 cells/cm) throughout the colon.^[2]^

It has been suggested that a pathological inflammatory response leads to ganglionic destruction and hence, the progress of acquired HG.^[8]^ Faussone-Pellegrini et al,^[9]^ proposed a theory of cellular structural remodeling. In patients with HG, a T cell-mediated response occurs first in the destruction of the intestinal nervous system. The resulting denervation stimulates a hypertrophic muscle response. This is one of the typical features of HG histology. Interstitial cells of Cajal (ICCs) are pacemakers of gut motility and act as the mediators of intestinal nerve control.^[10]^ The neurons release the natural ligand for c-kit, and the maintenance of the ICC phenotype depends on the signaling of c-kit. Since the infiltration of T lymphocytes leads to the death of neurons, this causes a reduction in c-kit signaling. The pre-existing ICCs in the patient are transformed into smooth muscle cells, which further contributes to the fibromuscular changes of the colonic circular muscle.

HG with a TZ is a particularly severe dysmotility. There are reported cases in both South Korea and Japan, but no Chinese cases have been described in the literature. Song et al,^[11]^ reported that the location of the TZ was least observed in the sigmoid colon. The majority of prior case reports of acquired HG were diagnosed in the setting of a dilated sigmoid region. Therefore, the case described in this report is highly unusual.

Surgery is the definitive treatment method for adult HG.^[12]^ The principle of pull-through surgery is to first remove all the intestinal segments of the low ganglion cells, and second to anastomose the normal innervated intestine and anal canal to provide long-term intestinal control.^[13]^ Surgical procedures developed for the treatment of childhood diseases have been applied to adults, and there is no significant difference in efficacy between the 2.^[14]^ Patients with acquired HG may require multiple surgeries because of the extent of the associated lesions. This procedure results in high postoperative morbidity and mortality in young children, while it achieves a good outcome in older subjects.^[15]^

For this case, we hypothesize that a long and repeated inflammatory process affected the sigmoid colon, resulting in immune-mediated destruction of ganglion cells in the myenteric plexus. Due to the accumulation of feces in the patient's intestines, we were unable to perform an enteroscopy and accurately evaluate the potential cause of inflammation. Syphilis infection in the intestine has been reported in a few cases,^[16,17]^ therefore the patient's history of syphilis should not be ignored. The patient was initially referred to several hospitals for severe intestinal distension, but no further surgery was performed. Influenced by prolonged dilation and inflammation, the patient's intestinal wall was extremely edematous, and there was a high risk of anastomotic leakage after end-to-end anastomosis. Due to the poor intestinal conditions, she was treated with segmental resection and formation of stoma in our hospital. The main obstructive mechanism was attributed to a narrowed TZ.^[18]^ Based on our experience, resection of TZ and enterostomy presents an effective remission strategy for patients with HG. The symptoms, including defecation difficulties and abdominal distension, have disappeared, and the patient is currently alive with no evidence of recurrence.

## 4 Consent

The patient has provided informed consent for publication of the case. Informed written consent was obtained from the patient for publication of this case report and accompanying images.

## Author contributions

**Conceptualization:** Zhiping Pan.

**Data curation:** Zhiping Pan, Luqiao huang.

**Methodology:** Zhiping Pan.

**Supervision:** Junhui Cui.

**Writing – original draft:** Zhiping Pan.

## References

[1] Yoshimaru K Taguchi T Obata S Immunostaining for Hu C/D and CD56 is useful for a definitive histopathological diagnosis of congenital and acquired isolated hypoganglionosis. Virchows Archiv 2017;470:679–85. 28424865 10.1007/s00428-017-2128-9

[2] Do MY Myung SJ Park HJ Novel classification and pathogenetic analysis of hypoganglionosis and adult-onset Hirschsprung's disease. Dig Dis Sci Dig Dis Sci 2011;56:1818–27. 21222160 10.1007/s10620-010-1522-9

[3] Taguchi T Masumoto K Ieiri S New classification of hypoganglionosis: congenital and acquired hypoganglionosis. J Pediatr Surg 2006;41:0–2051. 10.1016/j.jpedsurg.2006.08.004 17161202

[4] Dingemann J Puri P Isolated hypoganglionosis: systematic review of a rare intestinal innervation defect. Pediatr Surg Int 2010;26:1111–5. 20721562 10.1007/s00383-010-2693-3

[5] Holland SK Hessler RB Reid-Nicholson MD Utilization of peripherin and S-100 immunohistochemistry in the diagnosis of Hirschsprung disease. Mod Pathol 2010;23:1173–9. 20495540 10.1038/modpathol.2010.104

[6] Kim HJ Kim AY Lee CW Hirschsprung disease and hypoganglionosis in adults: radiologic findings and differentiation. Radiology 2008;247:428. 18430875 10.1148/radiol.2472070182

[7] Taguchi T Ieiri S Miyoshi K The incidence and outcome of allied disorders of Hirschsprung's disease in Japan: results from a nationwide survey. Asian J Surg 2015;40:29–34. 26216257 10.1016/j.asjsur.2015.04.004

[8] Holland-Cunz S Göppl M Rauch U Acquired intestinal aganglionosis after a lytic infection with varicella-zoster virus. J Pediatr Surg 2006;41:e29–31. 10.1016/j.jpedsurg.2005.12.060 16516611

[9] Faussonepellegrini MS Fociani P Buffa R Loss of interstitial cells and a fibromuscular layer on the luminal side of the colonic circular muscle presenting as megacolon in an adult patient. Gut 1999;45:775–9. 10517919 10.1136/gut.45.5.775 PMC1727704

[10] Rolle U Yoneda A Solari V Abnormalities of C-Kit-positive cellular network in isolated hypoganglionosis. J Pediatr Surg 2002;37:709–14. 11987084 10.1053/jpsu.2002.32259

[11] Song EM Kim JW Lee SH Colonic pseudo-obstruction with transition zone: a peculiar eastern severe dysmotility. J Neurogastroenterol Motil 2019;25:137–47. 30646485 10.5056/jnm18121 PMC6326194

[12] Qadir I Salick MM Barakzai A Isolated adult hypoganglionosis presenting as sigmoid volvulus: a case report. J Med Case Rep 2011;5:445–50. 21902826 10.1186/1752-1947-5-445 PMC3179760

[13] Holschneider AM Puri P Homrighausen LH Intestinal neuronal malformations (IND): clinical experience and treatment. In: Holschneider AM Puri P, editors. Hirschsprung's disease and allied disorders. Springer Berlin Heidelberg; 2008: 229–51.

[14] Miyamoto M Egami K Maeda S Hirschsprung's disease in adults: report of a case and review of the literature. J Nippon Med Sch 2005;72:113–20. 15940019 10.1272/jnms.72.113

[15] Lu W Xiao Y Huang J Causes and prognosis of chronic intestinal pseudo-obstruction in 48 subjects. Medicine 2018;97:e12150. 30200110 10.1097/MD.0000000000012150 PMC6133590

[16] Song SH Jang I Kim BS A case of primary syphilis in the rectum. J Korean Med Sci 2005;20:886–7. 16224168 10.3346/jkms.2005.20.5.886 PMC2779291

[17] Ijiri M Fujiya M Ueno N Syphilis infection throughout the whole gastrointestinal tract. Endoscopy 2016;48 (S 01):E338–9. 27741533 10.1055/s-0042-117715

[18] Do YS Myung SJ Kwak SY Molecular and cellular characteristics of the colonic pseudo-obstruction in patients with intractable constipation. J Neurogastroenterol Motil 2015;21:560–70. 26424041 10.5056/jnm15048 PMC4622139

